# Tree mortality and recruitment in secondary Andean tropical mountain forests along a 3000 m elevation gradient

**DOI:** 10.1371/journal.pone.0300114

**Published:** 2024-03-11

**Authors:** Jenny C. Ordoñez, Esteban Pinto, Antonella Bernardi, Francisco Cuesta

**Affiliations:** 1 Grupo de Investigación en Biodiversidad, Medio Ambiente y Salud -BIOMAS—Universidad de Las Américas (UDLA), Quito, Ecuador; 2 Department of Biological Sciences, Auburn University, Auburn, AL, United States of America; UNAM, MEXICO

## Abstract

This study addresses the understudied dynamics of mortality and recruitment in Tropical Mountain forests, critical determinants of forest structural processes and biomass turnover. We examine how these demographic processes change with elevation and varying degrees of forest recovery by utilizing two forest censuses (2015 and 2019) from 16 plots (0.36 ha) across a 600–3500 m asl elevation gradient in the Ecuadorian Andes. Employing multivariate PCA analyses, we characterize successional forest dynamics and explore relationships between demographic rates, elevation, and indicators of forest recovery using standard linear regression and generalized additive models (GAMs). Contrary to our hypothesis, mortality exhibits a unimodal response, peaking at mid-elevations, with no significant relationship to above-ground biomass productivity (AGBp). In our successional forests, dominance by fast-growing species alters expected patterns, leading to increased mortality rates and AGBp, particularly at low-mid elevations. Forest recovery emerges as a significant driver of mortality and the sole predictor of recruitment, especially across different recovery statuses. Although forest recovery doesn’t impact mortality rates, it elucidates the identity of declining species in forests with varying recovery degrees. Our findings underscore that while forest recovery does not alter mortality rates, it provides critical insights into understanding which species are affected under varying recovery conditions. Recruitment, primarily driven by successional dynamics, exhibits higher rates in sites with less recovery. Furthermore, we demonstrate the utility of forest structure indicators, such as above-ground biomass, in inferring successional dynamics when the time since the last disturbance is unknown. The study emphasizes the importance of considering disturbances in comprehending the intricate interplay between the environment and forest dynamics in secondary forests.

## Introduction

Tropical mountain forests (TMFs) in the Andes cover ~ 530.000 km^2^ in South America [1]. They are characterized by conspicuous changes in forest structure, plant composition, and functional and taxonomic diversity as elevation increases. Such changes create recognizable vegetation zones that harbor high biodiversity and provide essential ecosystem services for human populations [1]. At the same time, TMFs face a high risk of degradation by land use [2] and climate change [3].

The ecology of TMFs is less studied than that of tropical lowland forests or temperate forests, particularly regarding forest dynamics. At global and regional scales, mortality is positively related to net primary productivity (NPP) in mature forests [4, 5]. The mechanisms underlying this relationship are hypothesized to be trade-offs subject to natural selection, specifically growth vs. persistence and reproduction vs. persistence. In TMFs, generally, net primary productivity (NPP) [6] and above-ground biomass productivity (AGBp) [7] decrease at higher elevations compared to lower elevations but see Duque *et al*. [1] in forests where species of the Fagaceae family are present. Thus, mortality should also decrease with elevation coinciding with reductions in productivity [4]. Tree recruitment together with mortality determines changes in tree density and species composition but little is known about the potential effects of elevation on recruitment rates. Recent studies using stem-based estimates of mortality in the Andes have shown decreasing mortality with elevation [8–10]. Still, the negative relationship between mortality and elevation is not always consistent when mortality is based on tree stems [11] or tree biomass [1, 11, 12]. Such departures from expected patterns might result from differences in species composition or disturbance legacies, which are rarely considered [13].

TMFs in the Ecuadorian Andes have been subjected to disturbance since pre-colonial times [14, 15], including natural disturbances driven by topography, climate, and anthropic disturbances of different degrees from selective logging to transformation to agricultural land [2, 16, 17]. Under such conditions, TMFs form mosaics with different degrees of recovery over relatively short spatial and temporal scales [18]. Therefore, assessing mortality patterns in response to environmental gradients must explicitly include the effects of disturbance or, alternatively, the status of forest recovery. After a disturbance, forest structure and composition recover through changes in tree density, basal area, and above-ground biomass (AGB) accumulation [19]. In temperate and lowland tropical forests, there are common patterns of recovery in AGBp, demographic rates (e.g., mortality and recruitment), and tree growth [13, 20, 21] linked to shifts in the dominance of different plant strategies as time advances and light and soil nutrient conditions change. Specifically, AGB stock increase is primarily driven by longer wood residence times [7], whereas AGB growth rates, tree mortality, and recruitment decrease as forest age increases [22–24]. Likewise, tree mortality patterns shift altering tree size and density distributions. In secondary forests, mortality changes from density-dependent dynamics in younger forests, dominated by fast-growing species, where mortality is concentrated mainly on small trees due to strong competition (competitive thinning) to a mature thinning phase where mortality occurs in large-size classes, mostly pioneers that reach their mature size and die due to competition for soil nutrients which become scarcer. Mortality in mature forests becomes more stochastic, reflecting the composition of slow-growing species compared to the dynamic nature of secondary forests [25].

Using data on two censuses (4 years apart) of 16 permanent plots (0.36 ha) located across 3000 m of an elevational gradient in the equatorial Andes, we investigated forest demography dynamics, particularly mortality and recruitment rates of secondary Andean forests of around 30 to 35 years of age. We hypothesize that in these TMFs, mortality, recruitment rates and AGBp should decrease in higher, colder sites, reflecting a shift towards plant communities with low forest productivity that favor persistence and survival in limited-resource environments. We also assessed whether the degree of forest recovery could alter this pattern by increasing mortality rates in early successional forests, irrespective of their position along the elevation gradient.

## Methods

### Study area and forest census

This study was carried out at the ‘Pichincha long-term forest dynamics and carbon monitoring transect’, situated on the north-western slope of the Ecuadorian Andes [26]. The transect is composed of 27 permanent plots of 60 m x 60 m [27], distributed between 600–3500 m asl. This study used 16 plots with complete census information for 2015 and 2019 (S1 Appendix). The selected plots are spaced ~200 m along the elevation gradient and represent well the four main ecosystem types along the gradient [28]. Although our plots are relatively small, they form homogenous units in an ecosystem with high variability across small spatial scales [16], such conditions are well suited to study potential impacts of disturbances typical of Andean TMF [12]. Daily air and soil temperature data are available for all plots for the period 2016–2020 derived from two temperature sensors (TidbiT® v2 Temp Logger) that were installed in each plot in 2015 (one sensor was buried 10 cm below the ground surface, and the other was positioned 10 cm above the ground surface) and a HOBO Pro v2 Temp / RH sensor installed at 1 m above the ground surface [29]. Mean annual air temperature ranges from 21.6° C at the lower end of the transect (600 m asl) to 7.1° C at the upper end—increasing ca. 0.6° C per 100 m asl. For full detail on site description see [27]. There are no measured data on precipitation for the sites, but annual precipitation derived from the CHELSA dataset at 1 km2 [30] also declines with elevation, from 2771 mm at the lowest site to 1251 mm at the highest site. The sampled forest are humid evergreen ecosystems with limited or no seasonality.

In these forests, tree α and β diversity are high (more than 500 woody species have been identified) with many rare species, particularly in plots under 2000 m asl (Cuesta *et al*. in preparation). Moreover, the selected plots correspond to successional forests affected by human activities of cattle grazing and selective logging between the late 1980s and 1990s. Currently, these forests are located within conservation private reserves, which has allowed ca. 30–35 years of ongoing recovery, evidenced in the forest composition, structure, and canopy configuration [26].

Forest structural variables, biomass dynamics indicators, and demographic rates were estimated from census data. In each plot, we mapped (X and Y coordinates) and measured all trees, palms, and fern trees with ≥ 5 cm of diameter at breast high (DBH) or 1.30 m of height. We tagged all measured trees and tracked them through time. We estimated tree height (H), in meters visually, and 10% of the population within each plot was randomly measured with the use of clinometers (Suunto©). During the last census in 2019, we recorded DBH growth, recruitment, and mortality. In cases where the recorded DBH growth of the last census was less than −0.1 cm y^−1^ or greater than 7.5 cm y^−1^, the DBH recorded was augmented/reduced to match these minimum/maximum values to avoid over or under-estimations of (AGB) [31]. This study was conducted under scientific research authorization from the Ministry of Environment of Ecuador: N029-2019 IC-FLO-DNB/MA.

### Indicators of forest recovery status and forest recovery pathways

Because the exact time since abandonment and the intensity of the disturbance are not known for our forests, we characterized 1) forest recovery status using forest community indicators that are known to covary with forest age: basal area (BA in m^2^ ha^-1^) or AGB stocks (Mg ha^-1^) [19]; 2) forest recovery dynamics, in terms of the pathway that the sampled forests are following in their recovery, with a β parameter sensu Coomes and Allen [25] and 3) disturbance intensity based on the change of Endemism Richness (ER) [32], change between censuses (see below).

#### Above-ground biomass stock (AGB)

We estimated stem AGB during the first year of the census using a pantropical allometric equation [33], defined as:

$$AGB=0.0673\times {\left(WD\times DBH^{2}\times H\right)}^{0.976},$$

where AGB (kg) is the estimated AGB, DBH (cm) is the diameter of the tree at breast height, H (m) is the estimated total height, and WD (g cm^−3^) is the stem wood density. We derived wood density from global datasets [34] and AGB was estimated using the BIOMASS package [35]. Values for tree density, BA, and AGB obtained in sampled plots of 0.36 ha, were upscaled to one hectare.

#### β parameter

The framework of Coomes and Allen [25] characterizes forest recovery development in three phases: competitive thinning, mature thinning, and mature forests where disturbance starts again (i.e., disturbed forests). Changes in tree size and tree density distributions define these phases. During competitive thinning and mature thinning phases, DBH increases, and tree density decreases with time. In these initial stages of forest development, where tree density and productivity are high, there is increased tree mortality driven by competition. During competitive thinning, mortality occurs in small-size classes: trees that die due to asymmetric competition for light; and during mature thinning, mortality occurs in large-size classes, mostly pioneers that reach their mature size and die due to competition for soil nutrients which become scarcer. As forests mature, both mortality and recruitment rates decrease and converge until disturbances (natural or anthropogenic) initiate the cycle again, opening space for new recruits. At this stage, mortality is driven by the death of large stems and is not related to density-dependent processes. In disturbed forests tree density increases and DBH decreases. We used a size-dependent parameter of mortality (β) to differentiate plots along a gradient of disturbance that ranges from sites strongly influenced by competitive thinning post internal disturbance (low β) to sites more influenced by active disturbances (high β).

To estimate β values, we first assessed changes in average tree DBH and tree density between 2015 and 2019 for each plot to establish whether these forests were in a thinning or a disturbed phase. We then estimated β through logistic regression by marking which trees have died between the census period and estimating the chance of dying depending on the original DBH size. Within forests in a thinning phase, tree density decreases, and DBH increases; moreover, a negative β parameter implies that mortality occurs in smaller DBH-size classes (i.e., forests are in competitive thinning). In contrast, within forests in thinning phases, a close to zero or positive β implies that mortality occurs in the larger size classes (mature thinning). In disturbed forests, tree density increases, DBH decreases, and β is zero or positive. The evaluation of changes in DBH and tree density between censuses in our forests showed that, between 2015 and 2019, 15 out of 16 plots increased stem density, and 13 out of 16 decreased average DBH, which would be more common after small-scale disturbances occur in mature forests, (S2 Appendix). Still, βeta (size dependent mortality) was negative in 15 out of 16 plots, but only the most negative β were significant at p <0.05 (β = -0.11 to -0.055, n = 6 plots ranging from 632–2313 m asl), while the rest were non-significant at p <0.05 (β = -0.053 to -0.008, n = 8 plots ranging from 827–3507 m asl). β values were not related to elevation (Table 1). The framework of Coomes and Allen [25], assessed in monotypic forests, assumes most saplings recruit shortly after the initial disturbance, and community tree density decreases when trees die. The discrepancy between changes in tree density and β values in our forests suggests that our TMFs are progressing towards maturity. However, in these highly diverse forests, recruiting of saplings continues even during the thinning phases. Such a phenomenon has also been observed in successional tropical lowland forests [36]. Therefore, we decided not to make the first separation between thinning and disturbed forests based on changes in tree density. Instead, we used β values specifically as indicators of size-related mortality for the PCA analysis. We assessed whether these forests might comply with competitive, mature thinning, or disturbance in combination with the other indicators of forest recovery.

**Table 1:** Pearson correlation among indicators of forest recovery (status and pathway), disturbance intensity and elevation.

	AGB-stocks_2015_ (Mg ha^-1^)	ER index	ER index change	β parameter	AGB productivity (Mg ha^-1^ y^-1^)	Elevation (m asl)
AGB-stocks_2015_		**0.54**	0.24	0.01	0.36	**-0.50**
ER index	0.030		0.07	-0.07	0.41	**-0.86**
ER index change	ns	ns		0.29	0.13	0.07
β parameter	ns	ns	ns		-0.13	0.36
AGB productivity (Mg ha^-1^ y^-1^)	ns	ns	ns	ns		**-0.69**
Elevation (m asl)	0.049	0.000	ns	ns	0.003	

#### Endemism richness change

Endemism richness (ER) was proposed as an index that combines a measure of endemism and species richness [32], and it has been used to assess the biological value of a particular area [37]. It has also been shown that “endemism richness”, in contrast to species richness, shows more consistently the impacts of disturbance intensity on soil invertebrates [38] and other 5 taxa across land uses with differing disturbance intensities in the same area of our study [39]: higher endemism richness as land use intensity decreases. Also in tropical forests, species richness tends to increase as forests mature, up to the climax, when species richness decreases [13] and recovery of endemism in secondary forests increases in long time scales [40]. In this study, we use ER as an indicator of the intensity of the disturbance in the sampled forests, and specifically ER change to assess the pathway of forest recovery towards maturity.

To estimate ER, we used species frequency data (the number of plots in which a species is present). The ER index of a species is the reciprocal of the species’ occurrence across all samples within the range of occurrence of the species. Thus, any given species recorded in only one plot will have an ER index = 1, while for species recorded in many plots, the ER index will approach zero [32]. Finally, the endemism richness (ER) of a plot (i.e., a community) is the sum of the ER index of each species present in that sample. Therefore, communities with a larger share of endemic species will have higher ER values. Since there are distinct differences in tree community composition related to elevational shifts [29], we did not estimate endemism across the whole gradient (16 plots). Our forests have four distinct forest ecosystems along the elevation gradient, and our 16 plots are evenly divided across these four ecosystems (S3 Appendix). We first estimated the ER for each species within these four ecosystems, and then we estimated each plot´s ER index. Change in ER was estimated as the difference between the ER index in the last year of the census minus the ER index of the first census. Positive values imply that communities are gaining endemic species and increasing richness (i.e., forests with advanced recovery, from less intense disturbance). In contrast a negative value implies communities are becoming more even (i.e. less recovered or communities that were subject to more intense disturbances and hence less recovered). The ER change between censuses varied from a net loss in ER (-6.3), to a net ER gain of 10.6- The observed trend was not related to elevation (Table 1).

#### Correcting for potential impacts of elevation on forest recovery indicators

One critical issue to consider with all the proposed forest recovery indicators is indicators such as AGB stocks, and ER covary with elevation. Thus, before assessing any impacts of disturbance on demographic rates it is imperative to remove any potential impact of the elevational gradient on these indicators. To do so, we run 95 quantile regressions for endemism richness and AGB against elevation, assuming higher values of richness and AGB stocks represent forests closer to mature conditions. Then, we estimated the normalized residuals of each plot for the 95^th^ percentile response, assuming the upper line represents the maximum response for any given elevation under the conditions of the study, following boundary line approaches [41]. Since we do not have mature forests as a reference of what could be the reference richness and AGB stocks in our sampled forests, we assume that for any given elevation, the forests with the greatest endemism richness and AGB stocks represent the oldest (best recovered) forest in our sample. Likewise, at any given elevation, we defined the less recovered forest as the farthest away from the regression fit and with the greatest residual dispersal with respect to the *reference* forests. We used normalized residuals (between 0 and 1) as the indicators of the forest recovery status to avoid potential differences with larger residuals from the lower elevation plots since the magnitude of the differences could be greater at lower elevations simply because maximum AGB stocks or ER could reach higher values at low elevations compared to mid and high elevation plots.

The regressions between AGB-stocks_2015_ (first census) and the ER index against elevation were highly significant, and their normalized residuals were unrelated to elevation (Fig B, S3 Appendix). Therefore, for the multivariate analysis, we used the normalized residuals of AGB-stocks_2015_ and community ER index as indicators. β values and change in ER were used as indicators directly.

### Estimation of demographic rates and biomass productivity

To characterize demographic dynamics, we estimated stem-based mortality and recruitment rates. Both rates were expressed in percentage: mortality rates (m, % y^-1^) and recruitment rates (r, trees reaching 5 cm DBH, % y^-1^) and they were estimated as:

$$m=(1‐{\left[1‐(N_{0}—N_{f})/N_{0}\right]}^{1/t})\times 100$$

$$r=(1‐{\left[1‐(N_{r}/N_{f})\right]}^{1/t})\times 100$$

Where N_0_ = the number of stems alive in the initial census, N_f_ = the number of stems alive in the second census, N_r_ = the number of stems recruited between censuses, and t = time in years [42].

Finally, we also estimated indicators of forest productivity, to run a separate test of whether mortality was positively related to forest productivity in these forests, according to expectations from Stephenson *et al*. [4]. We could not estimate forest NPP due to the lack of estimates of biomass losses (e.g., litterfall, consumer, and volatile compounds), but we could use census data to estimate AGB productivity (AGBp: Mg ha^-1^ y^-1^). AGBp was estimated as:

$$AGBp=(AGB_{surv}+AGB_{r})/t$$

where AGB_surv_ = biomass increment produced by the growth of all trees that survived between censuses; AGB_r_ = AGB of stems recruited between censuses and t = time in years [7].

### Data analysis

#### Characterizing forest recovery

We used multivariate analysis (principal component analysis—PCA) to assess the relationship structure among forest recovery (AGB stocks) and disturbance intensity (ER) indicators and extracted components that summarize the recovery status of the 16 forest plots. We tested three PCA models combining variables of forest recovery status, pathway, and disturbance intensity variables. The PCA analyses were performed with the *prcomp* function in stats R [43], with scaled and centered variables. For subsequent analysis, we chose the model that best explained the variability in the data in the first two axes. As forest recovery indicators we used the PCA loadings of each plot in axes 1 and 2 of the best PCA model. We also defined a categorical indicator of forest type: since there was an evident division of forest plots along PCA1 and PCA2, we also ran a K-means clustering with Euclidean distances using the scores of the best PCA results. Based on the groups established by the K means clustering (2 groups) we created a categorical variable that described each forest recovery group. This categorical variable was also used as an independent variable in subsequent regression models of demographic rates (see below).

#### Assessing modulation of demographic rates by environment and forest recovery

To characterize the environmental, gradient we used elevation as an integrative indicator of environmental changes that covary with elevation. The relationships between demographic rates vs. indicators of the environmental gradient and indicators of forest recovery were assessed through bivariate linear regressions of mortality and recruitment vs. each environmental and forest recovery indicator. We also ran Generalized Additive Models (GAMS) to assess non-linear relationships between demographic rates vs. indicators of the environmental gradient. To comply with assumptions of linearity, mortality, and recruitment rates were log10 transformed before running all models (bivariate and multivariate linear models).

Subsequently, we run various multivariate linear models relating mortality and recruitment vs. different combinations of elevation and forest recovery indicators (i.e. PCA scores for PCA axes 1 and 2 and the category of forest type). We also assessed non-linear relationships in multivariate models using GAMS with a non-linear term for environmental indicators and linear effects for forest recovery indicators. These models were fitted with a reduced set of independents due to the small sample size, which limited the number of parameters to be tested (See S4 Appendix for details of all models tested).

Lastly, we assessed the relationship between AGBp vs. demographic rates to check whether the reduction in AGBp with elevation could also explain our expectation of decreasing demographic rates as elevation increases. If that were the case, mortality, and AGBp should have a positive relationship, confirming the effect of elevation on tree demography due to shifts in plant strategies related to growth and persistence. Linear and non-linear models of demographic rates vs. AGBp were applied separately from other models that included elevation, to avoid collinearity problems due to the strong relationship of AGBp with elevation. Linear models were performed with the *lm* function from the stats package in R, and GAMS models were run with the *gam* function from the *rom mgcv* package [43].

## Results

### Characterization of forest recovery

Community AGB-stocks_2015_ ranged from 96.59 to 318.08 Mg ha^-1,^ and AGBp ranged between 0.82 and 7.97 Mg ha^-1^ y^-1^. ER index per plot ranged from 11.5 to 81.5 among the 16 plots. All variables decreased with increasing elevation (S4 Tables 1 and 3 in S4 Appendix).

From the three multivariate models evaluated, the model with the highest variance explained in forest recovery indicators was the PCA model 3, which included normalized residuals of AGB-stocks_2015_, β values, and the absolute change in plot ER between censuses (Table 2).

**Table 2:** Summary of PCA models of forest recovery of 16 plots. For each model, we include the indicators used and the individual and cumulative variance explained in the first two axes.

PCA Model	Model 1	Model 2	Model 3
Forest recovery status variables	• AGB-stocks_2015_Normalized residuals of plot ER in 2015	• Normalized residuals of AGB stock in 2015 • Normalized residuals of plot ER in 2015	• Normalized residuals of AGB stock in 2015
Forest recovery pathway variables	• β values • Change in plot ER between censuses	• β values • Change in plot ER between censuses	• β values • Change in plot ER between censuses
Variance explained in 1^st^ axis	41.5%	43.3%	52.6%
Variance explained in 2^nd^ axis	25.2%	25.8%	26.2%
Cumulative variance in axes 1 and 2	66.6%	69.1%	78.8%

**Fig 1:**
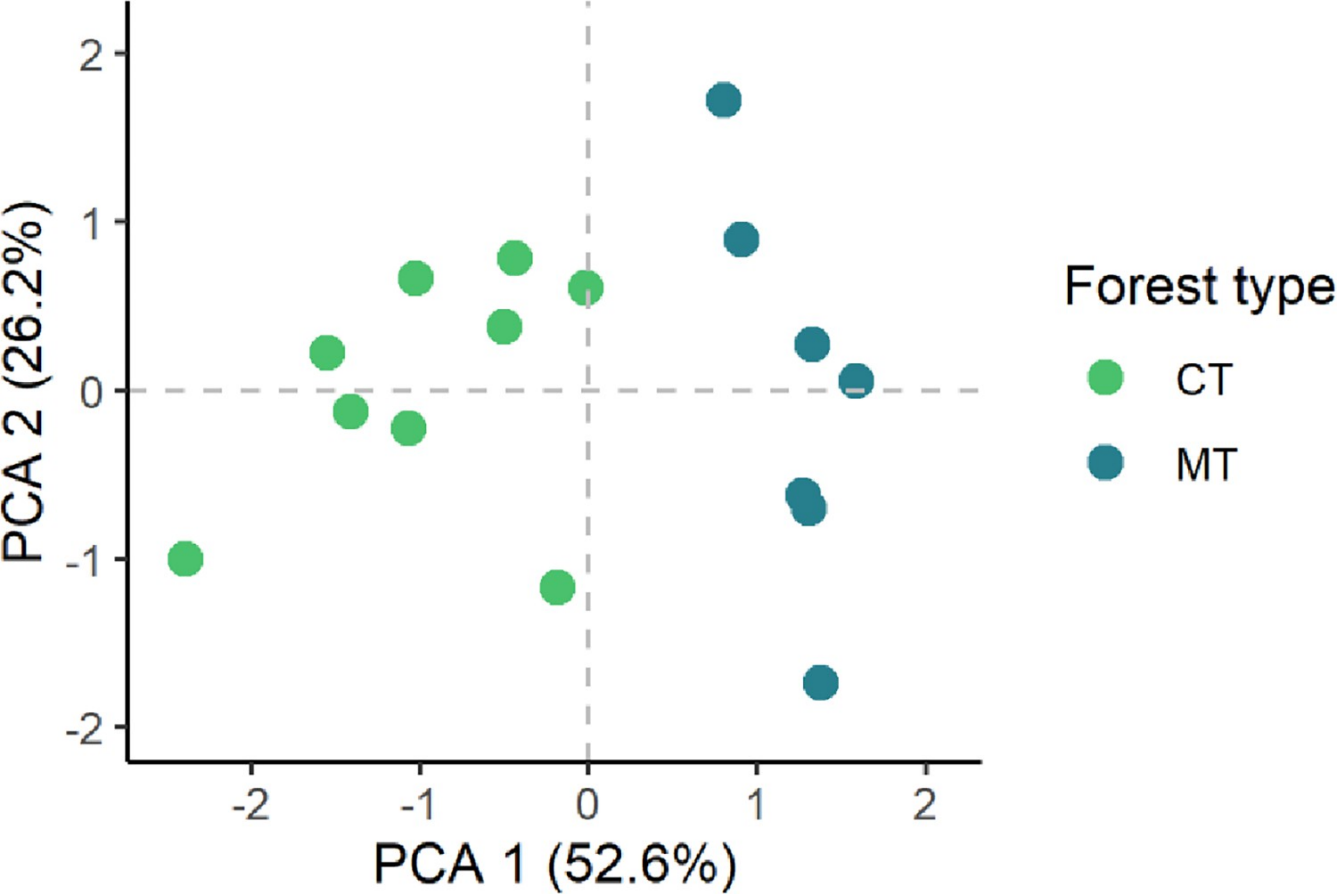
Principal component analysis (PCA) plot of sample scores (forest plots) across the first 2 PCA axes (variance explained in each axis between parenthesis). Colors represent two forest types identified by K-means clustering. CT = competitive thinning forests (green) and MT = mature thinning forests (blue).

The first axis of model 3 (PCA1) represented a forest recovery pathway: higher values in PCA1 were associated with forests with positive or close to zero β values, typical of mature forests that are entering a disturbed phase (*sensu* Coomes and Allen [25]). High PCA1 values were also associated with low residuals in AGB (i.e., forests closer to their maximum expected AGB stocks at a given elevation) and moderate increment in the ER index. At the lower end of the PCA1 axis, forests had negative β values typical of competitive thinning and high AGB stocks normalized residuals (forests far from the reference maximum AGB). The second axis (PCA 2) represented absolute changes in ER index. Higher values of PCA2 corresponded mainly to forests gaining endemic species (Table 3 and Fig 1), possibly reflecting forests under a fast recovery from less intense disturbances, and low values in PCA2, were associated with forests losing endemic species (Table 3 and Fig 1).

**Table 3:** PCA loadings for the first two axes of the best PCA model (PCA 3).

Variables	PC1	PC2
Normalized residuals of AGB stock in 2015	**-0.581**	**0.554**
Change in plot ER between 2019–2015	**0.530**	**0.814**
β values	**0.618**	-0.178

**Fig 2:**
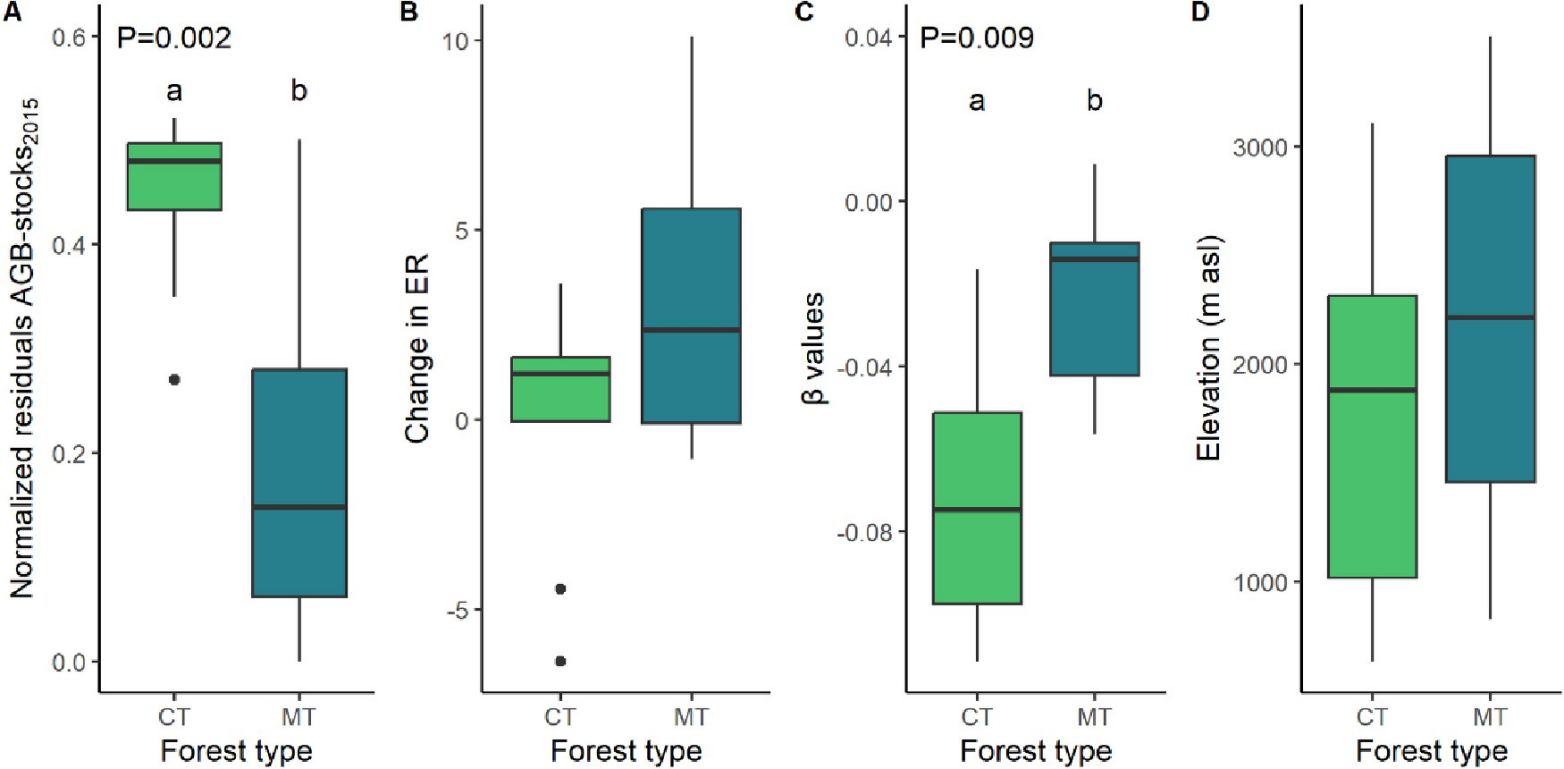
Boxplots of variables used as indicators of forest recovery in the best PCA model and elevation by forest recovery type. Colors represent two forest types identified by K-means clustering. CT = competitive thinning forests (green) and MT = mature thinning forests (blue). Significant differences between groups were tested with a 2-sided t-test.

K-means clustering identified two prominent forest types: Competitive Thinning Forests (CT), characterized by high AGB stock residuals and negative β values, and Mature Thinning Forests (MT), which exhibited an opposite trend, albeit with notable residual variability (Fig 1). Despite minor differences in ER change between the two groups (range of ER in MT forests = 0.4–10.7 and in CT forests = -6.3–3.7), we found a subtle difference. In MT Forests, ER increased as elevation decreased. In contrast, CT Forests exhibited ER increases in plots as elevation increased. Notably, both forest types effectively covered the entire elevation gradient (Fig 2).

### Modulation of demographic rates by elevation and forest recovery

**Fig 3:**
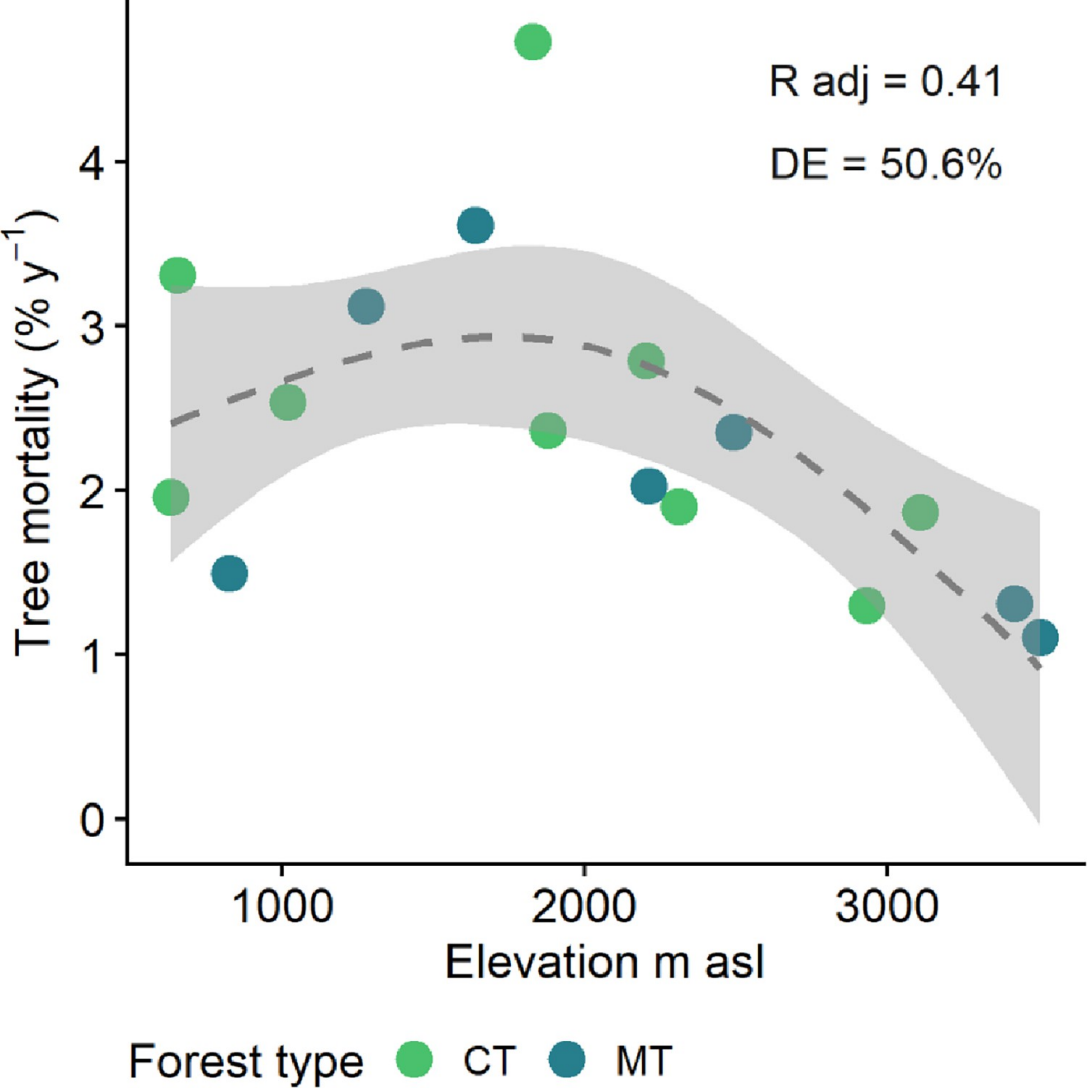
Best bivariate model: Tree mortality rates vs. elevation (GAMs parameters of the smooth term: EDF = 2.435, P = 0.038). Communities are colored by forest recovery type: CT = competitive thinning forests (green) and MT = mature thinning forests (blue), but GAM model has been fit for the whole dataset. DE = deviance explained.

The environmental gradient was the main driver of mortality rates across all forest plots in the bivariate models (Table A, S4 Appendix). Contrary to our hypothesis of decreasing rates with elevation, we observed a non-linear relationship between mortality and elevation with a quadratic form (EDF ~ 2). While mortality rates in low-warm sites (below 2000 m asl) were slightly higher (1.5–4.7% y^-1^) than in high-cold sites (> 2000 m asl) (1.1–2.8% y^-1^), the anticipated decrease with elevation did not emerge. Instead, mortality increased with elevation until reaching a peak around ~1800 m asl, beyond which it monotonically decreased (Fig 3). Recruitment rates, as revealed by bivariate linear or non-linear models, were not significantly related to environmental or forest recovery indicators.

When considering multivariate models of mortality using GAMS, it was not possible to model non-linear effects for all independents or their interactions due to the small sample size. Instead, we assessed different combinations of environmental gradient and forest recovery indicators (Table C, S4 Appendix). Most models increased the variance explained compared to bivariate relationships, but all models tested included non-significant terms at the P<0.05. Therefore, in this study, none of the multivariate GAM models for mortality were better than the bivariate GAM models with elevation.

The significance of the forest recovery indicator aligned with PCA axis1 only emerged in multivariate GAM models that distinguished between forest types (models 6 and 8 in Table D, S4 Appendix), rendering the effects of elevation non-significant. Consequently, we opted to evaluate multivariate models employing linear regression exclusively, focusing on forest recovery indicators. These models exhibited high significance regarding mortality and recruitment rates, elucidating distinct patterns for competitive thinning and mature thinning forests.

**Fig 4:**
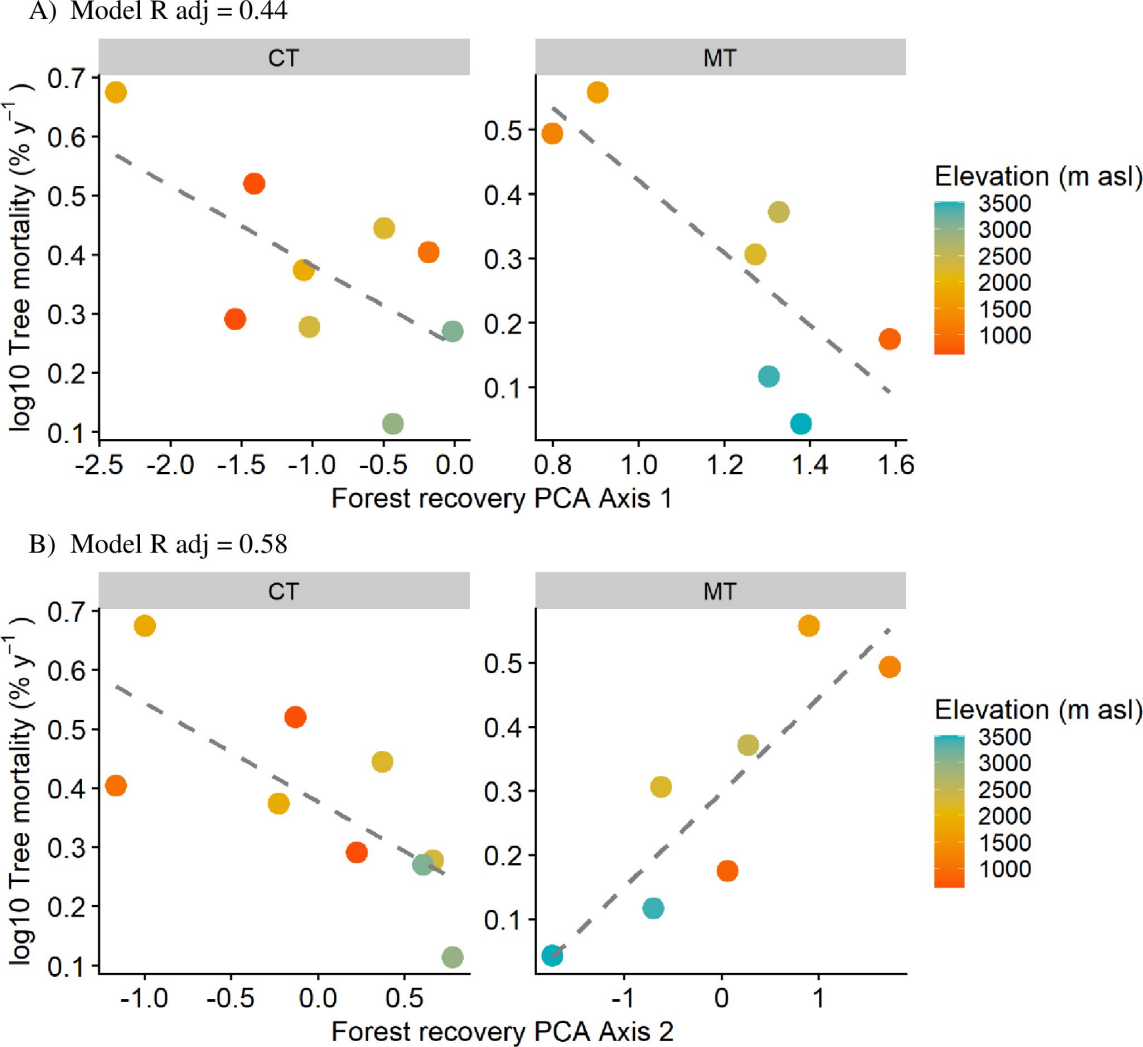
Multivariate models of mortality vs. A) PCA1 forest recovery and B) PCA2 endemism richness recovery. Models include interactions: varying intercepts and slopes by forest type: CT = competitive thinning forests; MT = mature thinning forests. Color gradient represents plot elevation in m asl.

Regarding mortality vs. PCA axis 1, both forest types exhibited a similar range of mortality rates. Within each forest type, higher PCA 1 values, exhibited lower mortality. However, the slopes were steeper in mature thinning forests, and intercepts were higher than in competitive thinning forests, resulting in a noticeable shift along the x-axis (Fig 4). The more recovered forests tended to be situated at higher elevations within each forest type. In the case of the relationship between mortality vs. forest type and richness recovery PCA2, we found a significant interaction between forest type and richness recovery PCA2. The interaction indicated that there was an opposite response of mortality with richness recovery PCA2 in competitive thinning and mature thinning forests (Table D, S4 Appendix). In competitive thinning forests, sites that increased ER had low mortality, while in mature thinning forests sites that increased in ER had high mortality (Fig 4).

Recruitment exhibited an exclusive association with forest recovery PCA1. The sole significant model for recruitment rates (R^2^adj = 0.54) incorporated forest recovery PCA1 and the categorical variable of forest recovery type (Table D, S4 Appendix). In this model, recruitment rates mirrored the mortality pattern: within each forest type, recruitment increased when values of PCA1 were low, albeit with distinct intercepts based on forest type. Specifically, mature thinning (MT) forests displayed higher intercepts, indicating elevated recruitment rates, compared to competitive thinning (CT) forests when forest recovery PCA1 scores were 0.

### AGB productivity and tree mortality along the elevation gradient

**Fig 5:**
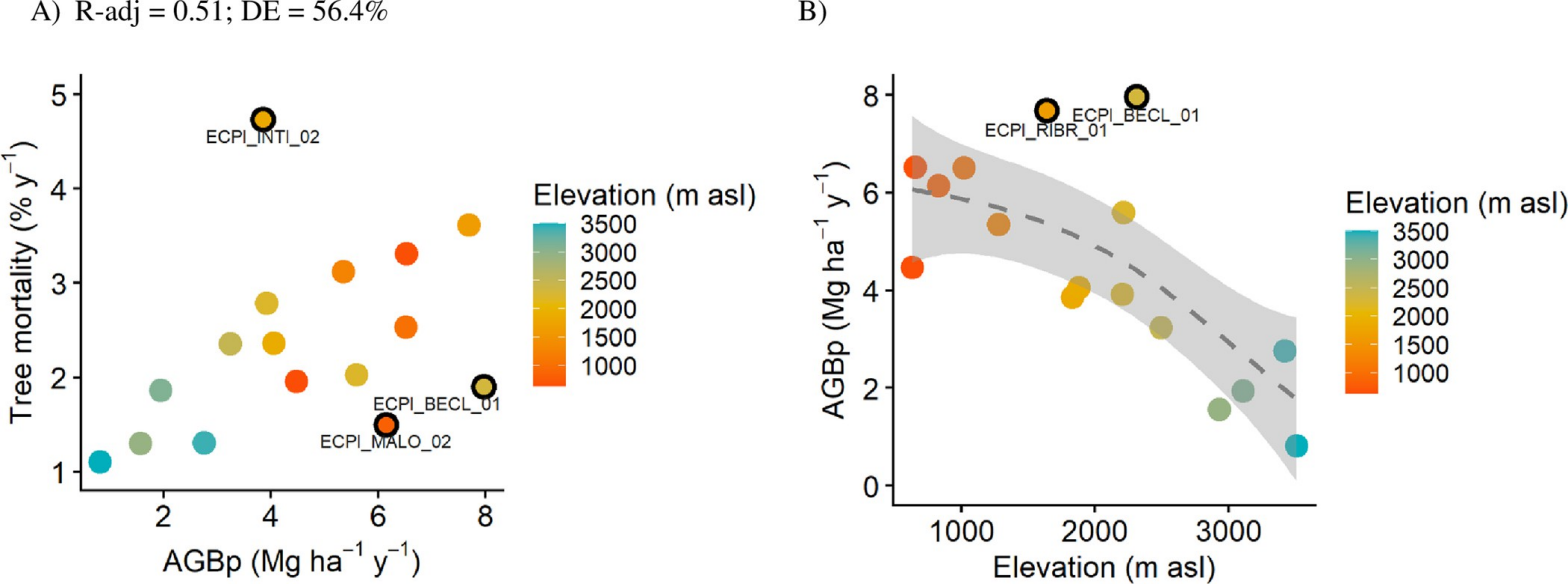
Relationship between Above-ground biomass productivity (AGBp) vs. elevation (A), and AGBp vs. tree mortality rates (B). Plots that deviate from the expected pattern are marked with a black border and plot name. DE = deviance explained.

AGBp decreased with elevation as expected. Both linear and non-linear models were highly significant. However, the non-linear relationship of AGBp and elevation had a better fit and was driven by extreme values corresponding to forest plots at intermediate elevations (Fig 5A).

Mortality rates showed a non-significant positive linear trend with AGBp (r = 0.40, p = 0.121). Further, non-linear relationships were not significant. The lack of significance in this relationship appeared to be influenced by three plots with notable deviations from the linear trend (see Fig 5B). Excluding these plots revealed a robust positive linear association between mortality and AGBp (r = 0.85, p = 0.000, Fig B, S4 Appendix).

## Discussion

We assessed the variation of demographic rates in naturally regenerated secondary forests of 30–35 years of age across a 3000 m elevation gradient in the western slope of the Equatorial Andes. Using the β parameter, the residuals of biomass stocks at the first census (2015), and changes in the ER index between censuses, we derived meaningful indicators of successional status and forest dynamics for our permanent plots. Our findings revealed a unimodal relationship between mortality and elevation, reaching a peak at mid elevations, while changes in AGBp were not significantly linked to mortality. The deviation from the expected demographic dynamics could be caused by disturbance effects leading to increased mortality and AGBp in specific plots. Forest recovery drove mortality and it was the only significant predictor of recruitment, particularly when separating forests with different recovery statuses. While forest recovery did not alter mortality rates, it provided valuable insights into forest demographic dynamics in successional forests with different degrees of recovery.

### Characterization of forest recovery using forest structural and diversity indicators

The absence of information about the time since the last disturbance poses a challenge to understanding successional forest dynamics. Our sampled TMFs corresponded to successional forests recovering from human activities from the late 1980s and early 1990s. We expected that after 30–35 years of natural regeneration, these forests would transition to the competitive or mature thinning phase, characterized by the death of suppressed trees and pioneer species, resulting in reduced tree density and increased DBH and height of surviving trees [25], our findings partially supported this expectation. Notably, tree densities increased, and DBH decreased in 15 out of 16 plots, which would be more common after small-scale disturbances occur in mature forests, but not under forest thinning. This discrepancy in our results may reflect continuous recruitment of species as forests mature, a phenomenon that has been reported in other tropical forests [36]. Nevertheless, combining β values with other indicators of recovery and disturbance intensity enabled a clear differentiation of two forest types consistent with successional theory [19, 44]: the less recovered forests (CT, n = 9) exhibited lower AGB-stocks_2015_ than expected, more negative β values implying that tree mortality is occurring mainly in smaller DBH size classes and little or diminishing ER index (~0, suggesting increasing homogeneity and reduced richness in terms of endemic species composition). Mature thinning forests (MT; n = 7) displayed AGB-stocks_2015_ close to the maximum expected under the study conditions, had β values close to 0 (implying mortality occurs in large DBH size classes), and an increasing ER index, signifying an increase in the number of endemic species.

### Impacts of elevation gradient on mortality rates

We hypothesized a negative relationship between tree mortality and increasing elevation. However, our findings revealed a non-linear pattern, with a peak in tree mortality at mid elevation (~1800 m asl) and a slight decline in mortality above 2000 m asl. Previous studies assessing demographic rates across elevation gradients in TMFs reported analogous non-linear response in Costa Rica [9] and a linear negative response in Argentina [8], albeit with considerable variability in their relationships. Although AGBp indeed decreased with elevation in our sampled TMFs, the relationship was non-linear, and we found no significant association between AGBp and mortality across our 16 forest plots. While mature forests exhibit a positive correlation between mortality and productivity [4], our results indicate that disturbance in secondary forests undergoing recovery alters these expected patterns. In all the relationships assessed, the deviation of our results from the hypotheses was driven by a few plots. These plots were located at low to intermediate elevations (three plots between 827–2330 m asl with very high mortality rates and two forest plots between 1640 and 2313 m asl with very high AGBp) where there seems to be better conditions for opportunistic and competitive species to thrive. The deviant plots corresponded to one plot with the highest mortality (INTI 2) and three with high productivity (i.e. BECL 1, RIBR 1, and MALO 2). The underlying reasons for the deviations were different for each case but in 3 out of 4 plots, the deviations seemed to be influenced by disturbance effects. In INTI 2, there was a strong dominance of palms (i.e., *Prestoea acuminata*) in the small size class (trees < 10 cm DBH) in the first year, and a great number of stems died between 2015 and 2019. These palm species are typical of disturbed habitats and are considered gap opportunists [45]. Palms’ extremely high mortality rates did not correspond with their overall contribution to AGBp: palms accounted for 9.9% of the AGBp, which corresponded almost entirely to biomass gained through natural regeneration. In BECL1 and RIBR 1, there was also a disproportionate contribution to productivity (57.3% in BECL1 and 33.9% in RIBR 1) by early and late pioneer species [46] from the Melastomataceae and Chloranthaceae families, such as *Axinaea quitensis*, *Hedyosmum cuatrecazanum*, *H*. *anisodorum*, *Miconia clathrantha*, and *M*. *theaezans*. Thus in 3 out of 4 plots, the influence of species with rapid opportunistic proliferation (e.g., palms) and fast growth altered the expected patterns. MALO 2 is a particular case since this is one of the plots better recovered in the whole altitudinal gradient. It has many large trees (DBH > 30) where the most contribution of AGB came from species in the Burseracea, and Violacea families but from species typical of mature forests such as *Dacryodes cupularis* and *Brosimum utile* subsp. o*ccidentale* [46].

For plots above 2000 m asl, there was a clearer convergence to low mortality rates and AGBp as elevation increased. This pattern might suggest that trade-offs between tree growth vs. persistence and reproduction vs. persistence play a role, where conservation strategies pay off by favoring tree survival over fast growth at high elevations. The shift in strategies from growth towards persistence has been observed in TMFs, where plant communities adapt to stress by narrowing strategies toward conserving resources at high elevations [27, 47, 48]. Unfortunately, the sample size of our study does not permit us to make strong generalizations in this respect. We recommend extending the sample size and gathering information on plant functional traits to be able to test this robustly. Such information is greatly needed to assess the potential implication in the long term for the recovery of TMFs, particularly for forests at low and mid-elevations. Forests at low and mid elevations could be exposed to slow recovery if they become trapped in a reinforcing loop of fast-growing species dying in large numbers and releasing space for the colonization of fast-growing species again [49]. Such risks have been reported in other studies [50].

### Impacts of forest recovery on mortality and recruitment rates

Forest recovery was more important for mortality within forest types (competitive and mature thinning) and was the only factor driving recruitment. Models that considered forest types and the PCAscores of PCA1 had better explanatory power than the models with the indicators of the environmental gradient, highlighting the role of disturbance on demographic rates.

We hypothesized that forests under a competitive thinning phase would have higher demographic rates irrespective of their location along the elevation gradient (i.e., less recovered forests should have lower AGB-stocks_2015_ and higher mortality and recruitment rates than forests under a mature thinning phase). However, we found no differences in intercept among forest successional phases between demographic rates and the environmental space across the elevation gradient. Our findings suggest that contrary to our expectations, mortality rates among competitive and mature thinning forests are similar. However, in CT forests, tree mortality occurs primarily in small DBH trees (more negative values), whereas in MT forests, tree mortality occurs in large DBH trees (no significant or close to 0 values). Furthermore, within forest types, forest structure plays an important role in shaping demographic rates independent of elevation. Better recovered forests (High PCA 1 axis) had lower mortality and recruitment rates than less recovered forests within each forest type.

The lack of response of recruitment to elevation was somewhat puzzling considering that mortality and recruitment rates were highly correlated in our forests (r = 0.85, P = 2.64e-05). Recruitment rates varied more than mortality along the elevation gradient. Although a great part of this variation was caused by extreme values, particularly in CT forests, the separation of forest types did not improve the recruitment relationship with the elevation gradient. Hernández Gordillo *et al*. [9] also found that elevation did not affect recruitment rates. Variation in recruitment was solely explained by forest recovery indicators, specifically PCA axis1. Moreover, the correlation of mortality and recruitment rates was much higher in MT forests (See, Fig C, S4 Appendix), perhaps reflecting the expected pattern of convergence of vital rates as forests mature [44]. Altogether these results show that the environmental gradient imposed by elevation does not seem to affect microsite conditions to which regeneration niche specialization of seedlings might respond to increase the chance of survival. Forest recovery is the main driver of recruitment probably through changes in microsite abiotic conditions as succession proceeds and the influence of life history traits [51].

### Further steps

Our study has a limited time between censuses and a small sample size; thus, expanding coverage in time and the number of sites is necessary to test the generality of declining mortality rates with elevation. Maintenance of forest monitoring through permanent plots is strongly required to assess the recovery of our studied forests after disturbance and the synergetic effects of ongoing climate change on these highly diverse ecosystems. Specifically, it is necessary to monitor changes in forest structure and species composition of recruits to assess whether forests are following a path toward maturity or maintaining the dynamics of early successional forests. Likewise, it is necessary to study whether warmer and wetter conditions could facilitate species migration with more competitive strategies to higher elevations. The migration of species could change demographic rates and successional dynamics at higher elevations in habitats with limited resources.

## Supporting information

S1 Appendix Location of the 16 permanent plots across the elevation gradient on the western slope of the equatorial Andes. (DOCX)

S2 Appendix Characterization of successional dynamics with the Β parameter. (DOCX)

S3 Appendix Estimation of community forest structure and forest endemism richness indicators. (DOCX)

S4 Appendix Linear and non-linear models tested for mortality and recruitment. (DOCX)

S5 Appendix Tree community (plot) data. (DOCX)
