# Supplementary material for: Tree mortality and recruitment in secondary Andean tropical mountain forests along a 3000 m elevation gradient
Source: PLoS One. 2024 Mar 11;19(3):e0300114. doi: 10.1371/journal.pone.0300114 (PMC10927132; doi:10.1371/journal.pone.0300114)
Supplement: S1 Appendix — (DOCX) [file pone.0300114.s001.docx]

# Title: Tree mortality and recruitment in secondary Andean tropical mountain forests along a 3000 m elevation gradient

Jenny C. Ordoñez^1¶*^, Esteban Pinto^2&^_,_ Antonella Bernardi^1&^, Francisco Cuesta^1¶*^

^1^Grupo de Investigación en Biodiversidad, Medio Ambiente y Salud -BIOMAS - Universidad de Las Américas (UDLA) Quito, Ecuador.

^2^Department of Biological Sciences, Auburn University, Auburn AL 36849-5407 USA.

# Supporting information

# S1: ****Location of the 16 permanent plots across the elevation gradient**** ****on the western slope of the equatorial Andes.****

*
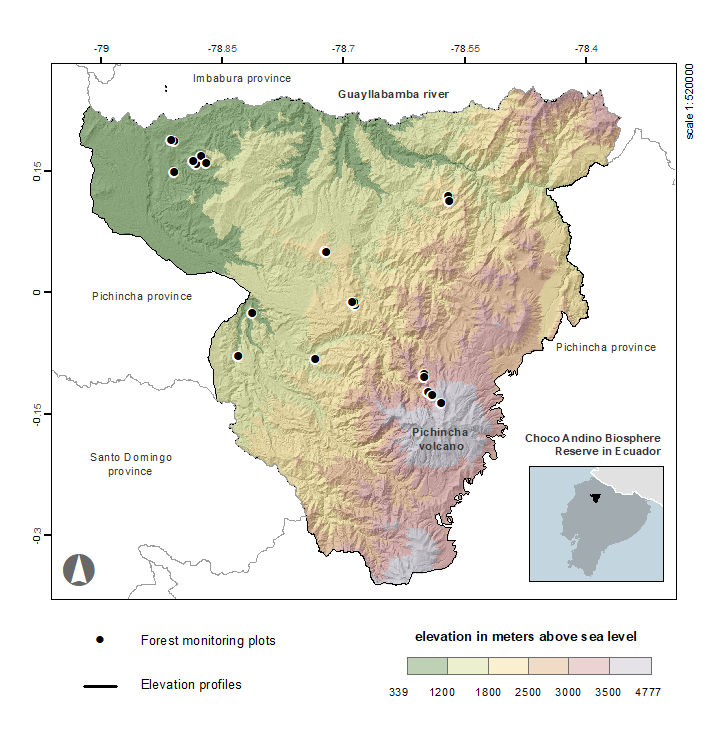
*
