# Supplementary material for: Tree mortality and recruitment in secondary Andean tropical mountain forests along a 3000 m elevation gradient
Source: PLoS One. 2024 Mar 11;19(3):e0300114. doi: 10.1371/journal.pone.0300114 (PMC10927132; doi:10.1371/journal.pone.0300114)
Supplement: S2 Appendix — (DOCX) [file pone.0300114.s002.docx]

# Title: Tree mortality and recruitment in secondary Andean tropical mountain forests along a 3000 m elevation gradient

Jenny C. Ordoñez^1¶*^, Esteban Pinto^2&^_,_ A. Bernardi^1&^, Francisco Cuesta^1¶*^

^1^Grupo de Investigación en Biodiversidad, Medio Ambiente y Salud -BIOMAS - Universidad de Las Américas (UDLA) Quito, Ecuador.

^2^Department of Biological Sciences, Auburn University, Auburn AL 36849-5407 USA.

# Supporting information

# S2. Characterization of successional dynamics with the Beta parameter

**Fig S2 A. Change in stem density and mean DBH for 16 forest communities in 4 years (2 census periods).** Circles correspond to the census in 2015 and triangles to the census in 2019. The elevation of each forest community is included in the figure as a color gradient.

| 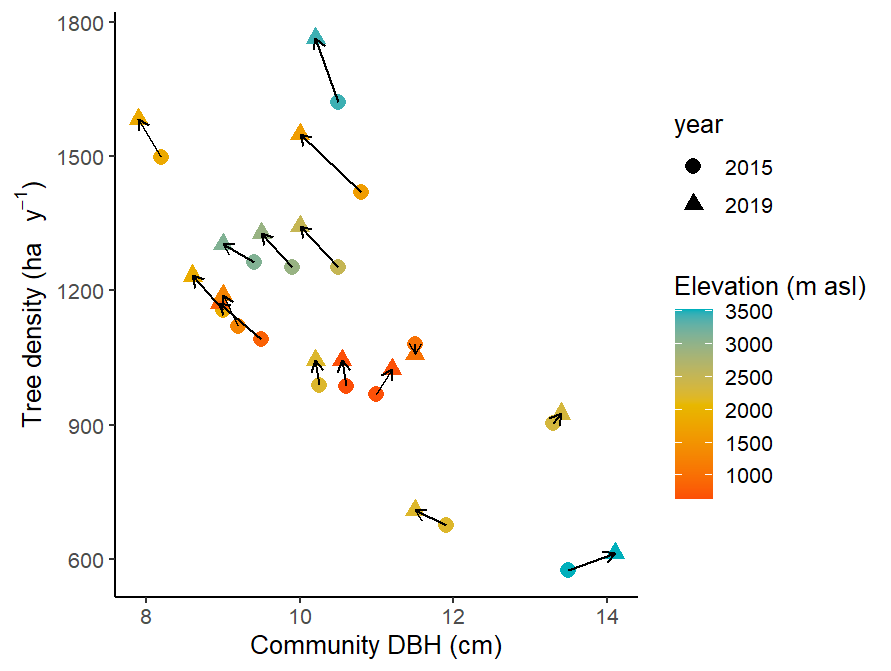 |
| --- |

**Table S2 A. Beta values, SE, and significance levels for 16 plots along the elevation gradient.**

| Plot ID | Elevation (m asl) | B value | S.E. Beta | P-value |
| --- | --- | --- | --- | --- |
| MAPI_02 | 632 | -0.087 | 0.041 | 0.035 |
| MAPI_01 | 653 | -0.075 | 0.028 | 0.009 |
| MALO_02 | 827 | -0.056 | 0.033 | 0.086 |
| MALO_01 | 1018 | -0.016 | 0.021 | 0.425 |
| MIND_01 | 1277 | -0.055 | 0.025 | 0.027 |
| RIBR_01 | 1640 | 0.009 | 0.019 | 0.631 |
| INTI_02 | 1829 | -0.099 | 0.026 | 0.000 |
| INTI_01 | 1879 | -0.111 | 0.039 | 0.004 |
| BECL_03 | 2203 | -0.051 | 0.030 | 0.088 |
| CEDR_03 | 2212 | -0.012 | 0.018 | 0.498 |
| BECL_01 | 2313 | -0.097 | 0.042 | 0.019 |
| CEDR_01 | 2492 | -0.029 | 0.020 | 0.144 |
| VERD_02 | 2932 | -0.053 | 0.040 | 0.180 |
| VERD_03 | 3109 | -0.033 | 0.026 | 0.206 |
| VERD_01 | 3421 | -0.014 | 0.027 | 0.597 |
| YANA_01 | 3507 | -0.008 | 0.025 | 0.746 |

**Fig S2 B. Distribution of trees with a DBH during the first year of the census (2015) > 30 cm (large trees) for A) CT forests and B) MT forests.**

| 1. **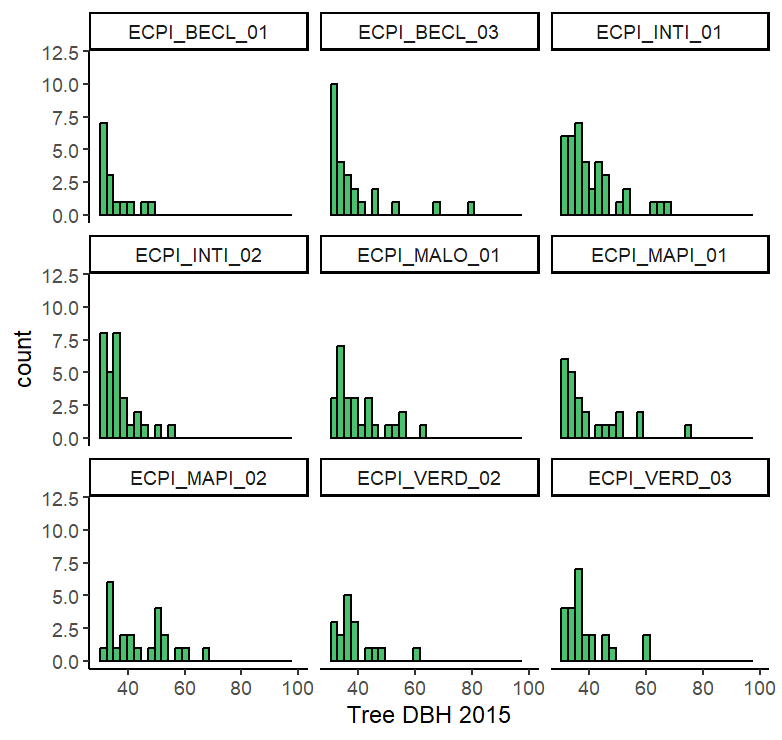** |
| --- |
| 1. **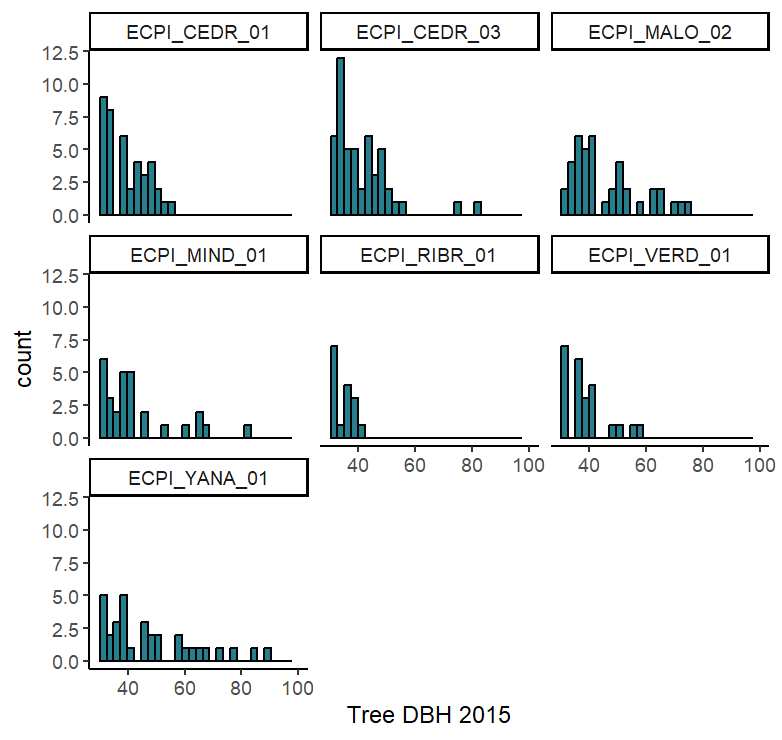** |
