# Supplementary material for: Tree mortality and recruitment in secondary Andean tropical mountain forests along a 3000 m elevation gradient
Source: PLoS One. 2024 Mar 11;19(3):e0300114. doi: 10.1371/journal.pone.0300114 (PMC10927132; doi:10.1371/journal.pone.0300114)
Supplement: S3 Appendix — (DOCX) [file pone.0300114.s003.docx]

# Title: Tree mortality and recruitment in secondary Andean tropical mountain forests along a 3000 m elevation gradient

Jenny C. Ordoñez^1¶*^, Esteban Pinto^2&^_,_ A. Bernardi^1&^, Francisco Cuesta^1¶*^

^1^Grupo de Investigación en Biodiversidad, Medio Ambiente y Salud -BIOMAS - Universidad de Las Américas (UDLA) Quito, Ecuador.

^2^Department of Biological Sciences, Auburn University, Auburn AL 36849-5407 USA.

# Supporting information

# S3. Estimation of community forest structure and forest endemism richness indicators.

## Definition of forest communities to estimate endemism richness.

Since plant composition varies with elevation, we first defined the main forest ecosystems in our elevation gradient for estimating endemism richness. We performed a non-metric multidimensional scaling analysis (NMDS) to define the ecosystems using species abundance data per plot.

**Fig S3 A. Clustering (A) and non-metric multidimensional scaling analysis (NMDS, B) of tree communities’ assemblages (plots) along an elevational gradient (600-3500 m asl) in the western slope of the Ecuadorian Andes.** The similarity distance used was Bray-Curtis with relative abundance scores of the species (square root transformed) as the main grouping criterion.

| 1. 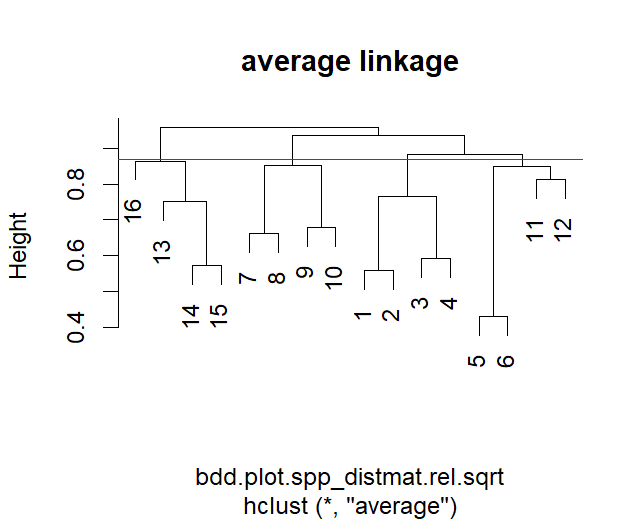 |
| --- |
| B)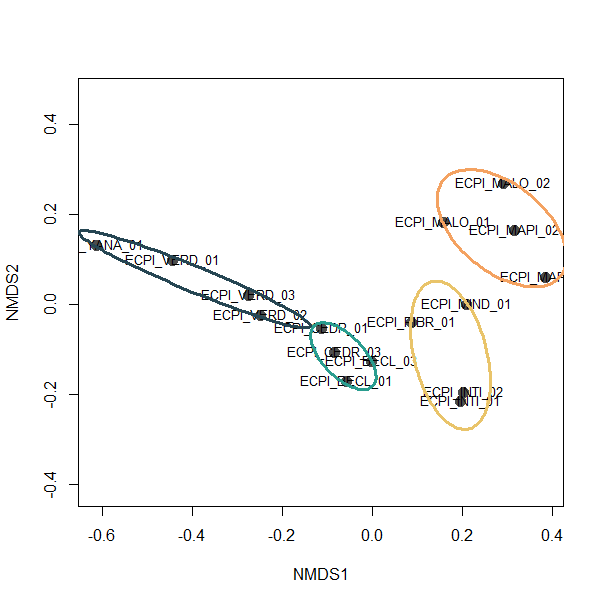 |

The NDMS was performed for 2 dimensions and reached a stress level of 0.074. We also performed a clustering analysis with the *hclust* function, using the dissimilarity matrix and average linkage method (Figure A2.1). We defined 4 groups that characterized well the division of the communities along the elevation gradient (Figure A2.2).

## Correcting for potential impacts of elevation on forest recovery indicators

We ran 95 quantile regressions for endemism richness (ER) and above-ground biomass in 2015 (AGB, Mg ha^-1^) against elevation, assuming higher values of richness and AGB stocks represent older, more recovered forests and forests recovering from a less intense disturbance event. Then, we estimated the normalized residuals of each plot with respect to this 95^th^-percentile response.

**Table S3 A. Quantile regression coefficients for the 95th percentile of AGB (Mg ha-1) and Community endemism richness vs. elevation.**

| Dependent variable | Intercept | Slope-elevation |
| --- | --- | --- |
| AGB (Mg ha^-1^) | 3.658e+02 | -5.776e-02 |
| ER | 1.059e+02 | -2.403e-02 |

**Fig S3 B. Quantile regressions for the 95th percentile of AGB stocks_2015_ (Mg ha^-1^) and community endemism richness (ER) vs. elevation (A and B).** AGB stocks_2015_ and community ER normalized residuals vs. elevation (C and D). Residuals were estimated as the difference between each observation and fitted values.

| A)  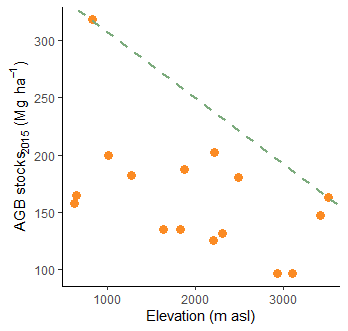 | B)  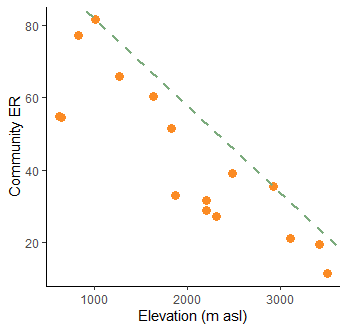 |
| --- | --- |
| C)  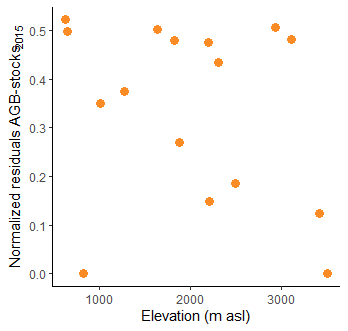 | D)  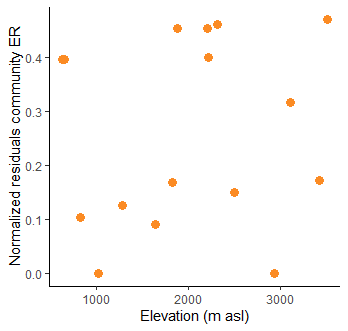 |
