# Supplementary material for: Tree mortality and recruitment in secondary Andean tropical mountain forests along a 3000 m elevation gradient
Source: PLoS One. 2024 Mar 11;19(3):e0300114. doi: 10.1371/journal.pone.0300114 (PMC10927132; doi:10.1371/journal.pone.0300114)

# Title: Tree mortality and recruitment in secondary Andean tropical mountain forests along a 3000 m elevation gradient

Jenny C. Ordoñez^1¶*^, Esteban Pinto^2&^_,_ A. Bernardi^1&^, Francisco Cuesta^1¶*^

^1^Grupo de Investigación en Biodiversidad, Medio Ambiente y Salud -BIOMAS - Universidad de Las Américas (UDLA) Quito, Ecuador.

^2^Department of Biological Sciences, Auburn University, Auburn AL 36849-5407 USA.

# Supporting information

# S4: Linear and non-linear models tested for mortality and recruitment.

We assessed the relationships of mortality (and recruitment) vs. each environmental and forest recovery indicator using linear regressions and Generalized Additive Models (GAMs) to test non-linear relationships. Since the relationship between mortality and the environmental indicators was non-linear, from here onwards, we present results of bivariate and multivariate models of mortality that include environmental indicators with GAMs. Linear regressions were used for multivariate models of mortality vs. forest recovery indicators as no evidence of non-linearity was observed in these models.

**Table S4 A. Bivariate non-linear regressions of mortality rates vs. environmental variables and forest recovery indicators using GAMs.**

| GAMs | EDF | F-statistic of smooth terms | P-value of smooth terms | Model Adj. R^2^ | Deviance explained |
| --- | --- | --- | --- | --- | --- |
| Elevation | 2.435 | 3.676 | 0.038 | 0.41 | 50.6% |

**Table S4 B. Bivariate linear regressions of mortality rates vs. forest recovery indicators.**

| Linear models | Effect direction | Model F-statistic | Model P value | Model Adj. R^2^ |
| --- | --- | --- | --- | --- |
| Independent variable | Mortality rates | | | |
| PCA1 forest recovery | - | 3.999 | 0.065 | 0.167 |
| PCA2 forest recovery | ns |  |  |  |
| PCA forest recovery type | ns |  |  |  |

**Table S4 C. Multivariate non-linear regressions of mortality rates vs. elevation and forest recovery indicators using GAMs. Elevation is modeled as a non-linear effect and forest recovery indicators as linear effects.**

|  |  | Linear effects | | Smooth terms | | | Model parameters | |
| --- | --- | --- | --- | --- | --- | --- | --- | --- |
| Model | Independent variable | Effect direction | Estimate P-value | EDF | F-statistic | Smooth P-value | Model Adj. R^2^ | Deviance explained |
|  |  |  |  |  |  |  |  |  |
| 1 | Elevation |  |  | 2.615 | 3.205 | 0.073 | 0.49 | 61.60% |
|  | PCA1 forest recovery | - | ns |  |  |  |  |  |
|  |  |  |  |  |  |  |  |  |
| 2 | Elevation |  |  | 2.368 | 3.404 | 0.048 | 0.37 | 51.40% |
|  | PCA2 forest recovery | - | ns |  |  |  |  |  |
|  |  |  |  |  |  |  |  |  |
| 3 | Elevation |  |  | 2.465 | 3.474 | 0.051 | 0.39 | 53.10% |
|  | PCA forest recovery type-MT | - | ns |  |  |  |  |  |
|  |  |  |  |  |  |  |  |  |
| 4 | Elevation |  |  | 2.206 | 1.832 | ns | 0.56 | 68.60% |
|  | PCA1 forest recovery | - | 0.023 |  |  |  |  |  |
|  | PCA forest recovery type-MT | + | 0.063 |  |  |  |  |  |
|  |  |  |  |  |  |  |  |  |
| 5 | Elevation |  |  | 2.358 | 3.005 | 0.068 | 0.34 | 53.30% |
|  | PCA2 forest recovery |  | ns |  |  |  |  |  |
|  | PCA forest recovery type-MT |  | ns |  |  |  |  |  |

**Table S4 D. Multivariate linear regressions of mortality and recruitment rates vs. forest recovery indicators.**

| Model | Independent variable | Effect direction | Estimate P-value | | Model F-statistic | | | Model P value | Model Adj. R^2^ | |
| --- | --- | --- | --- | --- | --- | --- | --- | --- | --- | --- |
|  |  |  |  | |  | | |  |  | |
| 6 | PCA1 forest recovery | - | 0.0205 | | 4.050 | | | 0.043 | 0.29 | |
|  | PCA forest recovery type-MT | + | 0.0876 | |  | | |  |  | |
|  |  |  |  | |  | | |  |  | |
| 7 | PCA2 forest recovery |  | ns | |  | | | ns |  | |
|  | PCA forest recovery type-MT |  | ns | |  | | |  |  | |
|  |  |  |  | |  | | |  |  | |
|  | PCA1 forest recovery | - | 0.049 | | 4.854 | | | 0.020 | 0.44 | |
| 8 | PCA forest recovery type-MT | + | 0.013 | |  | | |  |  | |
|  | PCA1 forest recovery *  PCA forest recovery type-MT | - | 0.059 | |  | | |  |  | |
|  |  |  |  | |  | | |  |  | |
| 9 | PCA2 forest recovery | - | 0.012 | | 8.047 | | | 0.003 | 0.58 | |
|  | PCA forest recovery type-MT | - | 0.189 | |  | | |  |  | |
|  | PCA2 forest recovery *  PCA forest recovery type-MT | + | 0.001 | |  | | |  |  | |
|  |  | Recruitment | | | | | | | |  |
| 1 | PCA1 forest recovery | - | | 0.001 | | 9.88 | 0.002 | | 0.54 |  |
|  | PCA forest recovery type-MT | + | | 0.001 | |  |  | |  |  |

**Fig S4 A. Multivariate models of recruitment (log-transformed) vs. PCA1 forest recovery.** The model includes an interaction term: varying intercepts and slopes by forest type: CT = competitive thinning forests; MT = mature thinning forests. Color gradient represents plot elevation in m asl.

| Model R adj = 0.54  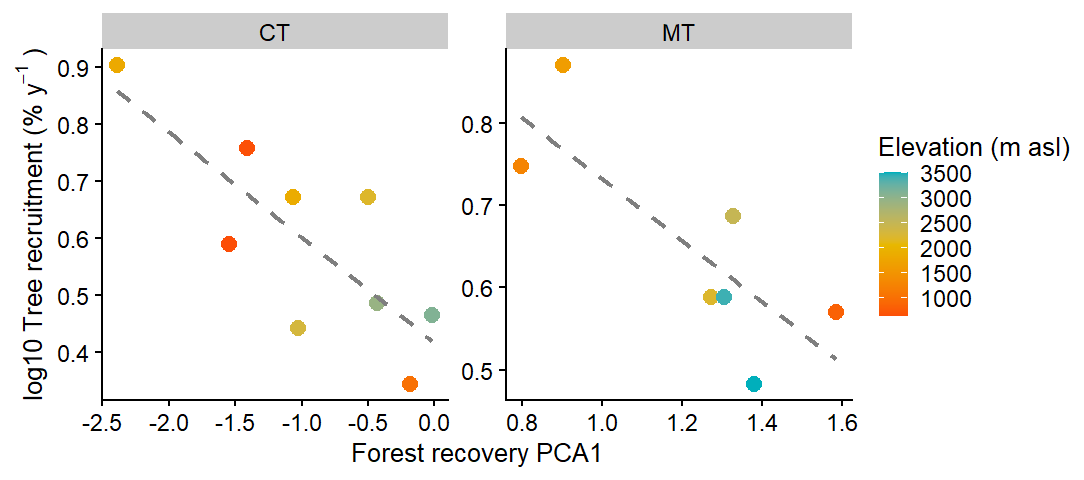 |
| --- |

**Fig S4 B. Relationship between AGBp vs. elevation (A) and AGBp vs. tree mortality rates (B**). Plots that deviate from the expected pattern have been excluded from the plot.

| *A)*  *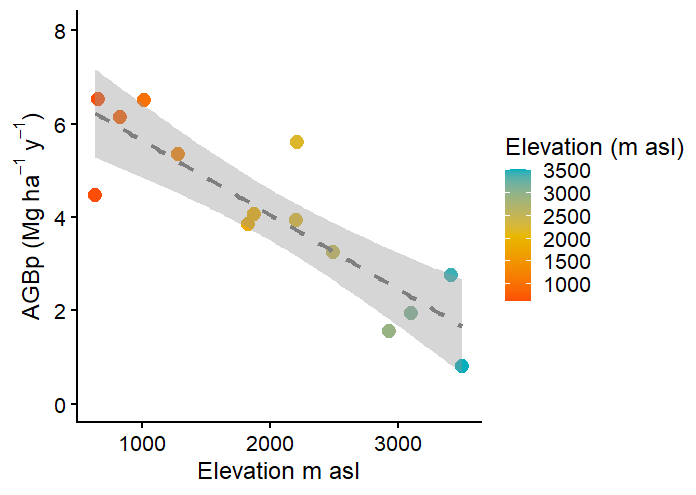* | *B)*  *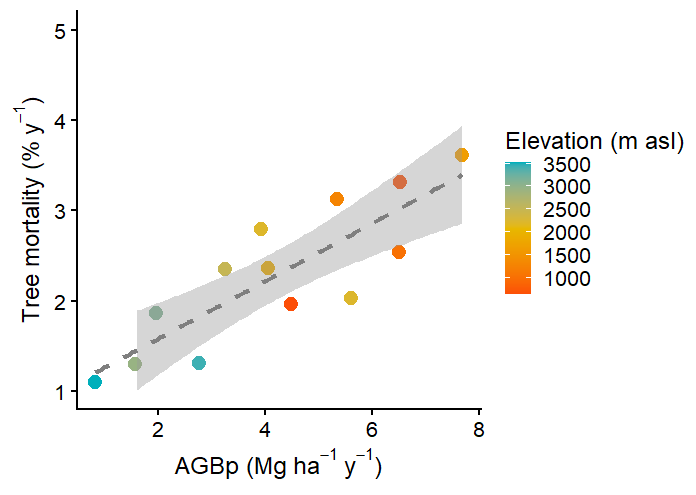* |
| --- | --- |

**Fig S4 C. Relationship between mortality and recruitment rates for CT forests (green) and MT forests (blue)**


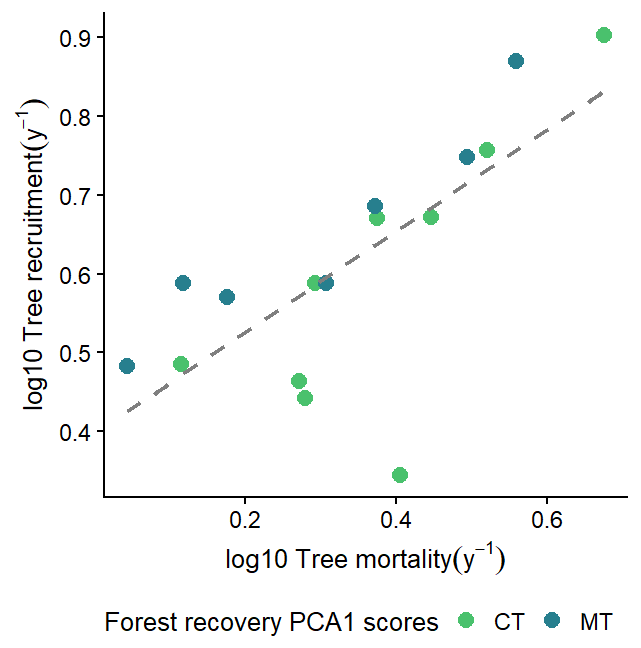

Supplement: S4 Appendix — (DOCX) [file pone.0300114.s004.docx]
