# Supplementary material for: Tree mortality and recruitment in secondary Andean tropical mountain forests along a 3000 m elevation gradient
Source: PLoS One. 2024 Mar 11;19(3):e0300114. doi: 10.1371/journal.pone.0300114 (PMC10927132; doi:10.1371/journal.pone.0300114)
Supplement: S5 Appendix — (DOCX) [file pone.0300114.s005.docx]

# Title: Tree mortality and recruitment in secondary Andean tropical mountain forests along a 3000 m elevation gradient

Jenny C. Ordoñez^1¶*^, Esteban Pinto^2&^_,_ A. Bernardi^1&^, Francisco Cuesta^1¶*^

^1^Grupo de Investigación en Biodiversidad, Medio Ambiente y Salud -BIOMAS - Universidad de Las Américas (UDLA) Quito, Ecuador.

^2^Department of Biological Sciences, Auburn University, Auburn AL 36849-5407 USA.

# Supporting information

# S5. Tree community (plot) data

**Table S5 A. Forest structure indicators for 16 plots from the census in 2015**

| Plot | Elevation | Minimum temperature (°C) | DBH (cm) | Tree density (trees ha^-1^) | Tree height (m) | Basal area 2015 (m^2^) | AGB  (Mg ha^-1^) | Endemism richness (ER) |
| --- | --- | --- | --- | --- | --- | --- | --- | --- |
| MAPI_02 | 632 | 22.23 | 10.6 | 986.1 | 12.1 | 22.1 | 157.63 | 54.83 |
| MAPI_01 | 653 | 22.23 | 11 | 969.4 | 12.5 | 21.8 | 164.90 | 54.50 |
| MALO_02 | 827 | 19.65 | 9.5 | 1091.7 | 11.0 | 31.7 | 318.08 | 77.17 |
| MALO_01 | 1018 | 18.79 | 11.5 | 1080.6 | 11.0 | 24.0 | 199.79 | 81.50 |
| MIND_01 | 1277 | 17.23 | 9.2 | 1119.4 | 10.0 | 25.1 | 182.71 | 65.83 |
| RIBR_01 | 1640 | 15.22 | 10.8 | 1419.4 | 10.1 | 22.3 | 135.22 | 60.50 |
| INTI_02 | 1829 | 14.46 | 8.2 | 1497.2 | 9.3 | 24.1 | 135.31 | 51.50 |
| INTI_01 | 1879 | 14.3 | 9 | 1155.6 | 9.0 | 28.0 | 187.71 | 33.17 |
| BECL_03 | 2203 | 12.13 | 11.9 | 675.0 | 8.4 | 18.0 | 125.46 | 29.00 |
| CEDR_03 | 2212 | 12.66 | 10.25 | 988.9 | 9.9 | 30.1 | 202.72 | 31.67 |
| BECL_01 | 2313 | 12.14 | 13.3 | 902.8 | 13.2 | 20.0 | 131.58 | 27.17 |
| CEDR_01 | 2492 | 11.38 | 10.5 | 1252.8 | 9.0 | 30.4 | 180.73 | 39.17 |
| VERD_02 | 2932 | 7.96 | 9.9 | 1252.8 | 10.7 | 21.1 | 97.00 | 35.50 |
| VERD_03 | 3109 | 7.98 | 9.4 | 1263.9 | 8.0 | 24.3 | 96.59 | 21.33 |
| VERD_01 | 3421 | 6.33 | 10.5 | 1622.2 | 7.1 | 28.9 | 147.35 | 19.67 |
| YANA_01 | 3507 | 5.21 | 13.5 | 575.0 | 11.1 | 27.1 | 163.27 | 11.50 |

**Table S5 B. Forest structure indicators for 16 plots from the census in 2019**

| Plot | Elevation | Minimum temperature (°C) | DBH (cm) | Tree density (trees ha^-1^) | Tree height (m) | Basal area 2015 (m^2^) | AGB  (Mg ha^-1^) | Endemism richness |
| --- | --- | --- | --- | --- | --- | --- | --- | --- |
| MAPI_02 | 632 | 22.23 | 10.6 | 1044.4 | 12.1 | 23.7 | 174.16 | 54.58 |
| MAPI_01 | 653 | 22.23 | 11.2 | 1025.0 | 12.7 | 24.6 | 186.92 | 53.08 |
| MALO_02 | 827 | 19.65 | 9.0 | 1172.2 | 10.5 | 33.5 | 339.13 | 84.92 |
| MALO_01 | 1018 | 18.79 | 11.5 | 1058.3 | 11.0 | 24.5 | 213.11 | 78.42 |
| MIND_01 | 1277 | 17.23 | 9.0 | 1188.9 | 9.9 | 26.9 | 201.70 | 76.50 |
| RIBR_01 | 1640 | 15.22 | 10.0 | 1550.0 | 9.5 | 23.9 | 158.73 | 66.50 |
| INTI_02 | 1829 | 14.46 | 7.9 | 1583.3 | 9.1 | 25.0 | 146.37 | 45.17 |
| INTI_01 | 1879 | 14.3 | 8.6 | 1233.3 | 8.7 | 29.7 | 203.05 | 33.83 |
| BECL_03 | 2203 | 12.13 | 11.5 | 708.3 | 8.1 | 19.1 | 136.73 | 31.17 |
| CEDR_03 | 2212 | 12.66 | 10.2 | 1044.4 | 9.9 | 31.6 | 216.41 | 34.67 |
| BECL_01 | 2313 | 12.14 | 13.4 | 925.0 | 13.3 | 23.1 | 162.95 | 30.33 |
| CEDR_01 | 2492 | 11.38 | 10.0 | 1344.4 | 8.8 | 30.9 | 190.73 | 45.83 |
| VERD_02 | 2932 | 7.96 | 9.5 | 1327.8 | 10.6 | 21.8 | 102.29 | 39.25 |
| VERD_03 | 3109 | 7.98 | 9.0 | 1302.8 | 7.8 | 24.8 | 103.29 | 25.08 |
| VERD_01 | 3421 | 6.33 | 10.2 | 1763.9 | 6.9 | 29.6 | 151.65 | 22.58 |
| YANA_01 | 3507 | 5.21 | 14.1 | 613.9 | 11.3 | 27.2 | 162.36 | 11.08 |

**Table S5 C. Demographic rates, above-ground biomass productivity (AGBp), and endemism richness (ER) change between 2015 and 2019 for 16 forest plots.**

| Plot | Elevation | Forest recovery type | N trees dead  (plot) | N trees recruited  (plot) | N trees survivors  (plot) | Recruitment rate (% y^-1^) | Mortality rate  (% y^-1^) | AGBp (Mg ha^-1^ y^-1^) | Change in ER |
| --- | --- | --- | --- | --- | --- | --- | --- | --- | --- |
| MAPI_02 | 632 | CT | 27 | 48 | 328 | 3.88 | 1.96 | 4.475 | -0.250 |
| MAPI_01 | 653 | CT | 44 | 64 | 305 | 5.72 | 3.31 | 6.525 | -1.417 |
| MALO_02 | 827 | MT | 23 | 52 | 370 | 3.72 | 1.50 | 6.144 | 7.750 |
| MALO_01 | 1018 | CT | 38 | 30 | 351 | 2.21 | 2.54 | 6.509 | -3.083 |
| MIND_01 | 1277 | MT | 48 | 73 | 355 | 5.59 | 3.12 | 5.347 | 10.667 |
| RIBR_01 | 1640 | MT | 70 | 117 | 441 | 7.42 | 3.62 | 7.688 | 6.000 |
| INTI_02 | 1829 | CT | 95 | 126 | 444 | 8.01 | 4.73 | 3.858 | -6.333 |
| INTI_01 | 1879 | CT | 38 | 66 | 378 | 4.68 | 2.37 | 4.057 | 0.667 |
| BECL_03 | 2203 | CT | 26 | 38 | 217 | 4.70 | 2.79 | 3.925 | 2.167 |
| CEDR_03 | 2212 | MT | 28 | 48 | 328 | 3.88 | 2.03 | 5.597 | 3.000 |
| BECL_01 | 2313 | CT | 24 | 32 | 301 | 2.77 | 1.90 | 7.970 | 3.167 |
| CEDR_01 | 2492 | MT | 41 | 74 | 410 | 4.85 | 2.35 | 3.241 | 6.667 |
| VERD_02 | 2932 | CT | 23 | 50 | 428 | 3.06 | 1.30 | 1.567 | 3.750 |
| VERD_03 | 3109 | CT | 33 | 47 | 422 | 2.91 | 1.86 | 1.948 | 3.750 |
| VERD_01 | 3421 | MT | 30 | 81 | 554 | 3.87 | 1.31 | 2.757 | 2.917 |
| YANA_01 | 3507 | MT | 9 | 23 | 198 | 3.04 | 1.11 | 0.819 | -0.417 |
